# Prediction of depth of invasion and lymph node metastasis in superficial pharyngeal cancer by magnifying endoscopy using the Japan Esophageal Society classification

**DOI:** 10.1002/deo2.151

**Published:** 2022-07-12

**Authors:** Hayato Yamaguchi, Takashi Kawai, Masakatsu Fukuzawa, Daiki Nemoto, Yasuyuki Kagawa, Shin Kono, Sakiko Naito, Hiroki Sato, Naoyoshi Nagata, Mitsushige Sugimoto, Kiyoaki Tsukahara, Takao Itoi

**Affiliations:** ^1^ Department of Gastroenterology and Hepatology Tokyo Medical University Tokyo Japan; ^2^ Department of Gastroenterological Endoscopy Tokyo Medical University Hospital Tokyo Japan; ^3^ Department of Otorhinolaryngology, Head, and Neck Surgery Tokyo Medical University Tokyo Japan

**Keywords:** JES classification, magnifying endoscopy, narrowband imaging, pharyngeal cancer, tumor thickness

## Abstract

**Backgrounds:**

The pharynx has no muscularis mucosae, so it is unclear whether diagnostic techniques used for the esophagus can be applied to the pharynx. This study investigated the usefulness of magnifying endoscopy with narrowband imaging using the Japan Esophageal Society (JES) classification for predicting the depth of invasion and lymph node metastasis (LNM) in pharyngeal cancer.

**Methods:**

A total of 123 superficial pharyngeal carcinoma lesions that had been observed preoperatively with magnifying endoscopy with narrowband imaging between January 2014 and June 2021 were analyzed. Predictors of subepithelial invasion (SEP) and LNM were sought based on endoscopic findings, including microvascular morphology, using the JES classification.

**Results:**

The lesions were divided into carcinoma in situ (*n* = 41) and SEP (*n* = 82). Multivariate analysis identified B2–B3 vessels (odds ratio [OR] 6.54, 95% confidence interval [CI] 1.74–24.61, *p* = 0.005) and a middle/large avascular area (OR 4.15, 95% CI 1.18–14.62, *p* = 0.027) as independent predictors of SEP. Significant predictors of LNM were protruding type, B2–B3 vessels, middle/large avascular area, SEP, venous invasion, lymphatic invasion, and tumor thickness > 1000 μm. Median tumor thickness increased significantly in the order of B1 < B2 < B3 vessels (B1, 305 μm; B2, 1045 μm; B3, 4043 μm; *p* < 0.001). The LNM rates for B1, B2, and B3 vessels were 1.6% (1/63), 4.8% (2/42), and 55.6% (10/18), respectively (*p* < 0.001).

**Conclusions:**

Magnifying endoscopy with narrowband imaging using the JES classification could predict the depth of invasion in superficial pharyngeal carcinoma. The JES classification may contribute to the prediction of LNM, suggesting that it could serve as an alternative to tumor thickness.

## INTRODUCTION

The advent of gastrointestinal endoscopy has increased the opportunities for gastrointestinal endoscopists to detect and treat not only cancer of the gastrointestinal tract but also pharyngeal cancer.^1^, ^2^, ^3^, ^4^, ^5^ There is a close relationship between tumor depth, lymph node metastasis (LNM), and prognosis, so preoperative assessment of tumor depth is very important when determining the treatment strategy for gastrointestinal cancer. Magnifying endoscopy with narrowband imaging (ME‐NBI) is useful for predicting the depth of gastrointestinal cancer because it can observe microvascular patterns.^6^, ^7^ The Japan Esophageal Society (JES) classification system is now widely utilized to detect changes in the intrapapillary capillary loop pattern when diagnosing the depth of esophageal squamous cell carcinoma by magnifying endoscopy.^8^, ^9^ However, despite recent advances in the diagnosis of gastrointestinal (including esophageal) cancers using magnifying endoscopy, there is still no method for the endoscopic diagnosis of depth of invasion in pharyngeal cancer. The pharynx has no muscularis mucosae, so it is unclear whether the diagnostic techniques used for the esophagus can be applied to the pharynx. The aim of this study was to investigate the usefulness of ME‐NBI using the JES classification for predicting the depth of tumor invasion in pharyngeal cancer.

## METHODS

### Patients and lesions

A total of 139 lesions in 108 consecutive patients who underwent endoscopic submucosal dissection or endoscopic laryngopharyngeal surgery for pharyngeal tumors at Tokyo Medical University Hospital between January 2014 and June 2021 were identified. Twelve lesions in 12 patients who did not undergo ME‐NBI, two lesions in two patients with low‐grade intraepithelial neoplasia, one lesion in one patient with liposarcoma, and one lesion in one patient with lymphoepithelial carcinoma were excluded. Hence, a total of 123 lesions in 92 patients in whom an endoscopic diagnosis of pharyngeal cancer was made with ME‐NBI using the JES classification were enrolled in the study. The pharyngeal cancers observed on ME‐NBI were divided into a carcinoma in situ (CIS) group and a subepithelial invasion (SEP) group. White light imaging, ME‐NBI, and pathological findings were analyzed retrospectively. Endoscopic findings were assessed by gastroenterologists who were blinded to the histopathological findings using endoscopic images stored in an endoscopic image filing system at the time of the surgery. Assessment with ME‐NBI was performed immediately before performing resection in cases undergoing endoscopic laryngopharyngeal surgery or endoscopic submucosal dissection. The evaluators were gastroenterologists (Hayato Yamaguchi and Yasuyuki Kagawa) who had more than 5 years of experience performing ME‐NBI and had examined more than 500 patients using ME‐NBI.

The study was approved by the Ethics Committee of Tokyo Medical University Hospital (approval number T2021‐0004) and conducted in accordance with the principles of the Declaration of Helsinki. Informed consent was obtained from all patients whose data were included in the study.

### Endoscopic procedures

Diagnostic ME‐NBI (GIF‐H260Z or GIF‐H290Z; Olympus, Tokyo, Japan) was performed under general anesthesia in all patients. Endoscopic resection was indicated for stage Tis, T1, T2, and T3 pharyngeal cancers but not for those with muscle invasion or stage T4 disease. First, a curved rigid laryngoscope (Nagashima Medical Instruments Co., Ltd., Tokyo, Japan) was inserted in the pharyngeal lumen. Before resecting the lesion, the endoscopist observed the lesion in non‐magnified white light imaging mode and magnified NBI mode using a magnifying endoscope. The extent and margins of the lesion were determined using the magnifying endoscope and iodine staining. A head and neck surgeon then inserted a curved electrosurgical needle knife (KD‐600; Olympus) via the mouth and applied marks that would secure an adequate surgical margin of approximately 5 mm from the edge of the unstained area. The gastroenterologist injected a mixture of saline, indigo carmine, and epinephrine into the subepithelial layer beneath the lesion. Submucosal dissection or endoscopic laryngopharyngeal surgery was then performed while maintaining traction using an orally inserted curved grasping forceps. All lesions were removed by en bloc resection.

### Prediction of tumor invasion using ME‐NBI with the JES classification

**FIGURE 1:**
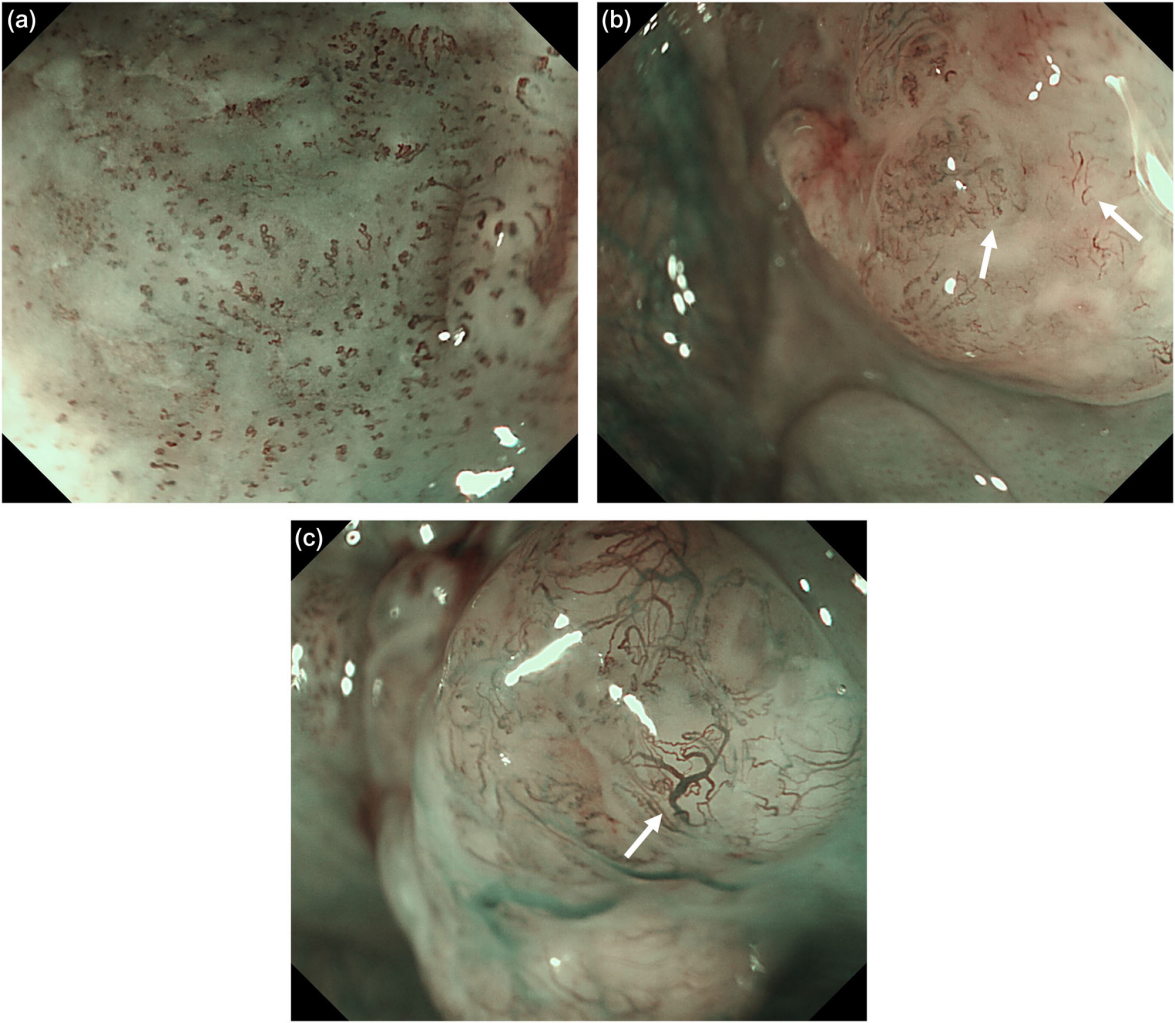
Categorization of type B vessels by magnifying endoscopy with narrowband imaging using the Japan Esophageal Society classification. Type B vessels were defined as abnormal microvessels with severe irregularity or highly dilated abnormal vessels. (a) B1, a vessel with a loop‐like formation (caliber approximately 20 μm). (b) B2, vessel without a loop‐like formation (white arrowhead). (c) B3, a highly dilated vessel with a caliber (often > 60 μm) that appears to be more than three times that of a typical B2 vessel (white arrowhead)

**FIGURE 2:**
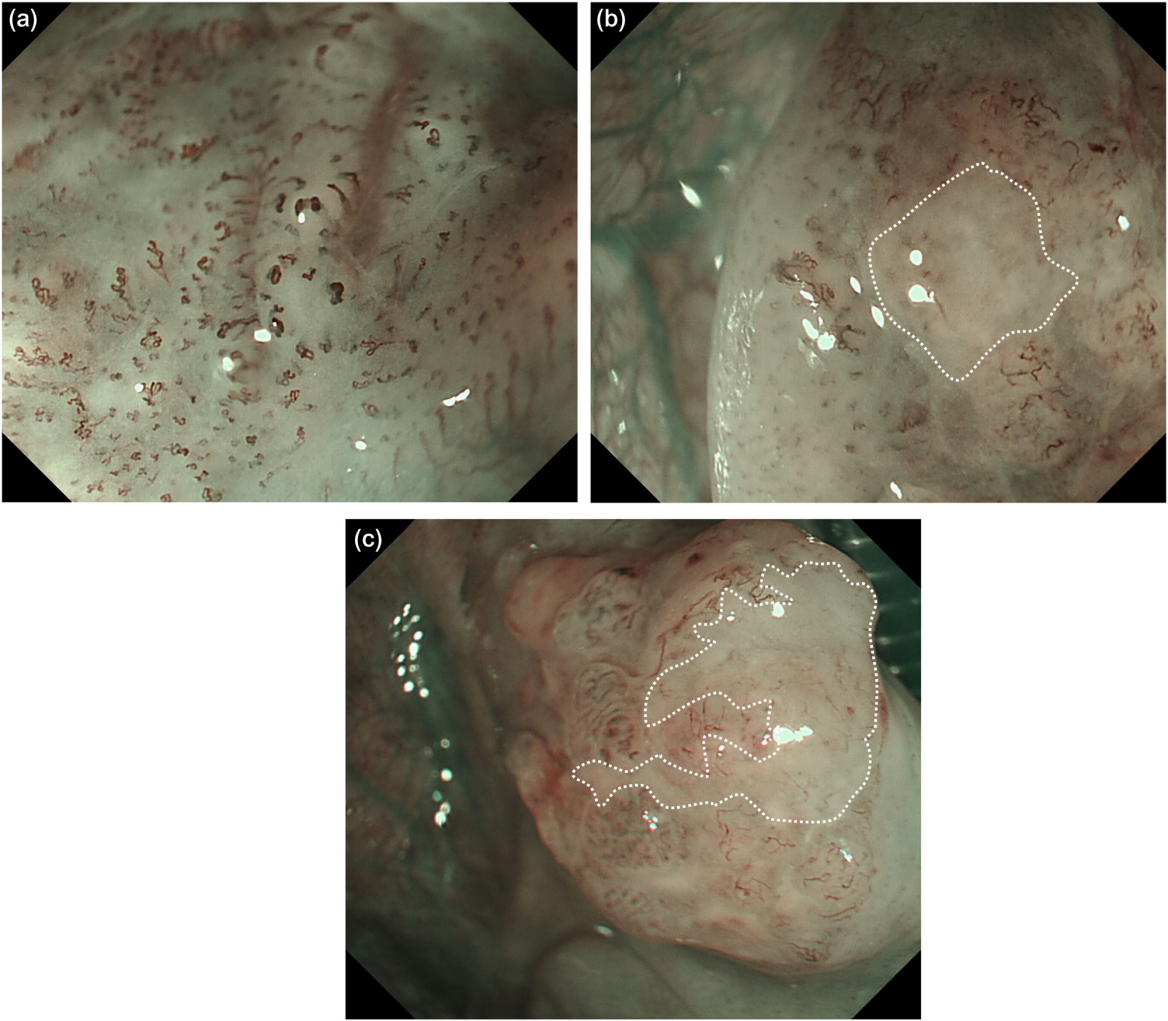
Categorization of the avascular area (AVA; defined as an area with low or no vascularity surrounded by type B microvessels) by magnifying endoscopy with narrowband imaging using the Japan Esophageal Society classification. (a) AVA‐small (<0.5 mm in diameter). (b) AVA‐middle (≥0.5 mm and ≤3 mm) (white dotted lines). (c) AVA‐large (≥3 mm) (white dotted lines)

The JES classification is a magnifying endoscopic diagnostic standard for superficial esophageal cancer devised by the JES.^10^ The microvessels of the lesion observed using magnifying endoscopy are categorized by degree of irregularity of weaving status, dilatation, caliber, and shape. Type B vessels are defined as abnormal microvessels with severe irregularity or highly dilated abnormal vessels and are classified as follows: B1, vessels with a loop‐like formation (caliber, approximately 20 μm); B2, vessels without loop‐like formation; B3, highly dilated vessels with a caliber more than three times that of typical B2 vessels (caliber often > 60 μm; Figure 1). An avascular area (AVA) was defined as an area of low or no vascularity surrounded by type B microvessels. The AVA was classified as small (AVA‐S, <0.5 mm in diameter), middle (AVA‐M, ≥0.5 and ≤3 mm), or large (AVA‐L, ≥3 mm; Figure 2).

Depth of pharyngeal cancer invasion was evaluated by ME‐NBI using the JES classification. A lesion was assumed to be CIS if it contained only B1 vessels or AVA‐S and to have reached the subepithelial layer or deeper if it contained B2/B3 vessels or AVA‐M/L.

### Evaluation of cervical LNM

Predictors of simultaneous cervical LNM were investigated based on endoscopic and pathological findings. Cervical LNM was evaluated by preoperative cervical ultrasonography, computed tomography (CT), magnetic resonance imaging, and positron emission tomography/CT (PET/CT). Patients with suspected LNM on these imaging modalities underwent fine‐needle aspiration cytology to determine whether there was a malignant component. When cervical LNM was confirmed by fine‐needle aspiration cytology, the patient underwent lymph node dissection. The neck was checked for LNM every 2–3 months by ultrasonography and CT and the chest for LMN every 6 months by CT. Simultaneous LNM was defined as LNM observed by cervical ultrasonography, CT, magnetic resonance imaging, or PET/CT before or within 1 year after endoscopic diagnosis using a magnifying endoscope.

### Histologic assessment

The resected specimens were fixed in 10% formalin, embedded in paraffin, cut into 2‐mm‐thick slices, and stained with hematoxylin‐eosin. CIS was defined as a lesion in which tumor cells were localized in the epithelial layer and SEP as a lesion in which tumor cells invaded the subepithelial layer. Tumor thickness was measured as the distance from the surface to the deepest point of the tumor.

### Statistical analysis

Results are presented as the median and interquartile range for continuous variables. Risk factors for tumor invasion of the subepithelial layer were analyzed using the Mann–Whitney *U*‐test for quantitative variables and Fisher's exact test or the chi‐squared test for categorical variables. Variables with a *p*‐value of <0.05 in univariate analysis were included in multiple logistic regression analysis. Tumor thickness was compared between the groups according to microvessel morphology using the Kruskal–Wallis test. All statistical analyses were performed using the SPSS software program (version 28; IBM Corp., Armonk, NY, USA). A *p*‐value < 0.05 was considered statistically significant.

## RESULTS

### Patient and lesion characteristics

Patient and lesion characteristics are shown in Table 1. The median age was 69 (63–73) years. The median tumor size was 19 (13–25) mm, and the macroscopic type was 0‐I in 26 lesions, 0‐IIa in 47 lesions, 0‐IIb in 38 lesions, and 0‐IIc in 12 lesions. Of the 123 lesions, 41 were pathologically diagnosed as Tis, 37 as T1, 38 as T2, and seven as T3. Microvascular morphology on ME‐NBI was classified as follows: B1/B2/B3 63/42/18 and AVA‐S/AVA‐M/AVA‐L 62/40/21. In terms of depth of invasion, 41 lesions were diagnosed as CIS and 82 as SEP.

**TABLE 1:** Patient and lesion characteristics

Patients (*n*)	92
Age, years, median (IQR)	69 (63–73)
Sex (male/female)	86/6
Body‐mass‐index, median (IQR)	22.0 (19.5–23.6)
Alcohol intake (no or mild/moderate/heavy)^a^	27/29/36
Smoking (no or mild/heavy)^b^	28/64

Lesions (*n*)	123
Tumor size, mm, median (IQR)	19 (13–25)
Tumor location, *n* (%)	
Hypopharynx	93 (75.6)
Pyriform sinus	53 (43.1)
Postcricoid	15 (12.2)
Posterior wall	25 (20.3)
Oropharynx	23 (18.7)
Anterior wall	8 (6.5)
Posterior wall	9 (7.3)
Lateral wall	6 (4.9)
Larynx	7 (5.7)
Supraglottic	7 (5.7)
Tumor morphology (0‐I/0‐IIa/0‐IIb/0‐IIc)	26/47/38/12
Type B vessels (B1/B2/B3)	63/42/18
AVA (small/middle/large)	62/40/21
T classification (Tis/T1/T2/T3)	41/37/38/7
Depth of tumor invasion (CIS/SEP)	41/82
Tumor thickness, μm, median (IQR)	486 (284–1600)
Venous invasion	12 (9.8)
Lymphatic invasion	13 (10.6)

Abbreviations: AVA, avascular area; CIS, carcinoma in situ; IQR, interquartile range; SEP, subepithelial invasion.

^a^ Alcohol: no or mild, < 10 g/day; moderate, ≥20 g and < 30 g/day; heavy, ≥30 g/day.

^b^ Smoking: no or mild, Brinkman index < 400; heavy, Brinkman index ≥400.

### Predictors of SEP

Predictors of SEP are shown in Table 2. All lesions were categorized as CIS or SEP. In univariate analysis, protruding‐type lesions (*p* < 0.001), B2–B3 vessels (*p* < 0.001), and AVA‐M/L (*p* < 0.001) were significantly more common in the SEP group than in the CIS group. There was no significant difference in age, sex, body mass index, tumor location or size, redness, white coat, or erosion between the CIS group and SEP group. Multivariate analysis identified B2–B3 vessels (odds ratio [OR] 6.54, 95% confidence interval [CI] 1.74–24.61, *p* = 0.005) and AVA‐M/L (OR 4.15, 95% CI 1.18–14.62, *p* = 0.027) to be independent predictors of SEP.

**TABLE 2:** Univariate and multivariate analyses of potential predictors of subepithelial invasion in patients with pharyngeal cancer

			Univariate analysis		Multivariate analysis	
Predictive factor	CIS (*n* = 41)	SEP (*n* = 82)	OR (95% CI)	*p*‐value	OR (95% CI)	*p*‐value
**Factors associated with patient's characteristics**						
Age, years, *n* (%)						
< 65	17 (41.5)	28 (34.1)	1.00 (reference)			
≥65	24 (58.5)	54 (65.9)	1.37 (0.63–2.95)	0.427		
Sex, *n* (%)						
Male	39 (95.1)	78 (95.1)	1.00 (reference)			
Female	2 (4.9)	4 (4.9)	1.00 (0.18–5.70)	0.654		
Body mass index, *n* (%)						
< 25	37 (90.2)	74 (90.2)	1.00 (reference)			
≥25	4 (9.8)	8 (9.8)	1.00 (0.28–3.54)	0.615		
**Factors associated with non‐magnified WLI findings**						
Tumor location, *n* (%)						
Hypopharynx or oropharynx	37 (90.2)	79(96.3)	1.00 (reference)			
Larynx	4 (9.8)	3(3.7)	0.35 (0.08–1.65)	0.167		
Tumor size, mm, *n* (%)						
< 40 mm	40 (97.6)	75 (91.5)	1.00 (reference)			
≥40 mm	1 (2.4)	7 (8.5)	3.73 (0.44–31.42)	0.186		
Tumor morphology, *n* (%)						
Flat type (0‐IIa/0‐II/0‐IIc)	40 (97.6)	57 (69.5)	1.00 (reference)		1.00 (reference)	
Protruded type (0‐I)	1 (2.4)	25 (30.5)	17.54 (2.28–134.82)	**<0.001**	4.08 (0.44–37.66)	0.215
Redness, *n* (%)						
Negative	29 (70.7)	47 (57.3)	1.00 (reference)			
Positive	12 (29.3)	35 (42.7)	1.80 (0.81–4.02)	0.149		
White coat, *n* (%)						
Negative	34 (82.9)	57 (69.5)	1.00 (reference)			
Positive	7 (17.1)	25 (30.5)	2.13 (0.83–5.45)	0.11		
Erosion, *n* (%)						
Negative	31 (75.6)	51 (62.2)	1.00 (reference)			
Positive	10 (24.4)	31 (37.8)	1.88 (0.81–4.37)	0.137		
**Factors associated with magnified NBI findings**						
Type B vessels, *n* (%)						
B 1	37 (90.2)	26 (31.7)	1.00 (reference)		1.00 (reference)	
B 2 + B 3	4 (9.8)	56 (68.3)	19.92 (6.43–61.78)	**<0.001**	6.54 (1.74–24.61)	**0.005**
AVA, *n* (%)						
AVA‐S	36 (87.8)	26 (31.7)	1.00 (reference)		1.00 (reference)	
AVA‐M + L	5 (12.2)	56 (68.3)	15.51 (5.46–44.08)	**<0.001**	4.15 (1.18–14.62)	**0.027**

Abbreviations: AVA, avascular area; CI, confidence interval; CIS, carcinoma in situ; NBI, narrowband imaging; OR, odds ratio; SEP, subepithelial invasion; WLI, white light imaging.

### Diagnostic value of ME‐NBI

The diagnostic value of ME‐NBI for estimating SEP and tumor thickness is shown in Table 3. In the SEP group, the sensitivity, specificity, positive predictive value (PPV), negative predictive value (NPV), and diagnostic accuracy of type B2–B3 vessels for diagnosing tumor invasion were 68.3%, 90.2%, 93.3%, 58.7%, and 75.6%, respectively; the respective values for AVA‐M/L were 68.3%, 87.8%, 91.8%, 58.1%, and 74.8%. The sensitivity, specificity, PPV, NPV, and diagnostic accuracy of type B2–B3 vessels for detection of tumor thickness ≥1000 μm were 93.2%, 75.9%, 68.3%, 95.2%, and 82.1%, respectively; the respective values for AVA‐M/L were 81.8%, 68.4%, 59.0%, 87.1%, and 73.2%.

**TABLE 3:** Diagnostic value of magnifying endoscopy with narrowband imaging for estimating subepithelial invasion and tumor thickness

Diagnosis for SEP invasion	Sensitivity (95% CI)	Specificity (95% CI)	PPV (95% CI)	NPV (95% CI)	Accuracy (95% CI)
Type B2 + B3	68.3 (57.1–78.1)	90.2 (76.9–97.3)	93.3 (83.8–98.2)	58.7 (45.6–71.0)	75.6 (67.0–82.9)
AVA‐M + L	68.3 (57.1–78.1)	87.8 (73.8–95.9)	91.8 (81.9–97.3)	58.1 (44.8–70.5)	74.8 (66.2–82.2)

Diagnosis for tumor thickness ≥1000 μm	Sensitivity (95% CI)	Specificity (95% CI)	PPV (95% CI)	NPV (95% CI)	Accuracy (95% CI)
Type B2 + B3	93.2 (81.3–98.6)	75.9 (65.0–84.9)	68.3 (55.0–79.7)	95.2 (86.7–99.0)	82.1 (74.2–88.4)
AVA‐M + L	81.8 (67.3–91.8)	68.4 (56.9–78.4)	59.0 (45.7–71.4)	87.1 (76.1–94.3)	73.2 (64.4–80.8)

Abbreviations: AVA, avascular area; CI, confidence interval; NPV, negative predictive value; PPV, positive predictive value; SEP, subepithelial invasion.

### Predictors of cervical LNM

Predictors of cervical LNM are shown in Table 4. Of the 123 lesions, 13 (10.6%) were positive for LNM and 110 (89.4%) were negative. In univariate analysis, significant predictors of LNM were protruding‐type morphology (*p* < 0.001), B2‐B3 vessels (*p* < 0.001), AVA‐M/L (*p* = 0.001), SEP (*p* = 0.004), venous invasion (*p* = 0.007), lymphatic invasion (*p* < 0.001), and tumor thickness > 1000 μm (*p* < 0.001).

**TABLE 4:** Univariate analysis of potential predictors of cervical lymph node metastasis in pharyngeal cancer

Predictive factor	LNM (−) (*n* = 110)	LNM (+) (*n* = 13)	OR (95% CI)	*p*‐value
**Factors associated with patient's characteristics**				
Age, years, *n* (%)				
<65	41 (37.3)	4 (30.8)		
≥65	69 (62.7)	9 (69.2)	1.34 (0.39–4.61)	0.447
Sex, *n* (%)				
Male	104 (94.5)	13 (100.0)		
Female	6 (5.5)	0 (0.0)	(incalculable)	0.504
Body mass index, *n* (%)				
<25	101 (91.8)	10 (76.9)		
≥25	9 (8.2)	3 (23.1)	3.37 (0.78–14.49)	0.116
**Factors associated with non‐magnified WLI findings**				
Tumor location, *n* (%)				
Hypopharynx or oropharynx	103 (93.6)	13 (100.0)		
Larynx	7 (6.4)	0 (0.0)	(incalculable)	0.448
Tumor size, mm, *n* (%)				
<40 mm	104 (94.5)	11 (84.6)		
≥40 mm	6 (5.5)	2 (15.4)	3.15 (0.57–17.54)	0.200
Tumor morphology, *n* (%)				
Flat type (0‐IIa/0‐IIb/0‐IIc)	92 (83.6)	5 (38.5)		
Protruded type (0‐I)	18 (16.4)	8 (61.5)	8.18 (2.40–27.87)	**<0.001**
Redness, *n* (%)				
Negative	67 (60.9)	9 (69.2)		
Positive	43 (39.1)	4 (30.8)	0.69 (0.20–2.39)	0.396
White coat, *n* (%)				
Negative	83 (75.5)	8 (61.5)		
Positive	27 (24.5)	5 (38.5)	1.92 (0.58–6.37)	0.222
Erosion, *n* (%)				
Negative	75 (68.2)	7 (53.8)		
Positive	35 (31.8)	6 (46.2)	1.84 (0.58–5.87)	0.230
**Factors associated with magnified NBI findings**				
Type B vessels, *n* (%)				
B 1	62 (56.4)	1 (7.7)		
B 2 + B 3	48 (43.6)	12 (92.3)	15.50 (1.95–123.39)	**<0.001**
AVA, *n* (%)				
AVA‐S	61 (55.5)	1 (7.7)		
AVA‐M + L	49 (44.5)	12 (92.3)	14.94 (1.88–118.90)	**0.001**
**Pathological findings**				
Depth of tumor invasion, *n* (%)				
CIS	41 (37.3)	0 (0.0)		
SEP	69 (62.7)	13 (100.0)	(incalculable)	**0.004**
Venous invasion, *n* (%)				
negative	102 (92.7)	9 (69.2)		
positive	8 (7.3)	4 (30.8)	5.67 (1.43–22.53)	**0.007**
Lymphatic invasion, *n* (%)				
negative	103 (93.6)	7 (53.8)		
positive	7 (6.4)	6 (46.2)	12.61 (3.32–47.80)	**<0.001**
Tumor thickness, *n* (%)				
<1000 μm	79 (71.8)	0 (0.0)		
≥1000 μm	31 (28.2)	13 (100.0)	(incalculable)	**<0.001**

Abbreviations: AVA, avascular area; CI, confidence interval; CIS, carcinoma in situ; LNM, lymph node metastasis; NBI, narrowband imaging; OR, odds ratio; SEP, subepithelial invasion; WLI, white light imaging.

### Relationship between ME‐BI findings and depth of invasion

The relationship between ME‐NBI findings and depth of invasion is shown in Table 5. B1 vessels were detected in 63 lesions, B2 vessels in 42, and B3 vessels in 18. In the SEP group, B1, B2, and B3 vessels were found in 41.3% (26 lesions), 90.5% (38 lesions), and 100% (18 lesions), respectively. The median tumor thickness of lesions with B1, B2, and B3 vessels was 305, 1045, and 4042.5 μm, respectively. The respective LNM rates for B1, B2, and B3 vessels were 1.6% (one lesion), 4.8% (two lesions), and 55.6% (10 lesions). Significant correlations were observed between type B vessels and tumor depth, tumor thickness, and LNM (*p* < 0.001). AVA‐S, AVA‐M, and AVA‐L were involved in 63, 40, and 21 lesions, respectively. In the SEP group, the AVA‐S, AVA‐M, and AVA‐L rates were 41.9% (26 lesions), 87.5% (35 lesions), and 100% (21 lesions), respectively; the respective median tumor thickness values were 331, 888.5, and 3500 μm and the respective LNM rates were 1.6% (one lesion), 15.0% (six lesions), and 28.6% (six lesions). A significant correlation was found between AVA and tumor depth, tumor thickness, and LNM (*p* < 0.001).

**TABLE 5:** Relationship between findings on magnified endoscopy with narrowband imaging and depth of invasion

Type B vessels	B1 (*n* = 63)	B2 (*n* = 42)	B3 (*B* = 18)	*p‐*value
SEP, *n* (%)	26/63 (41.3)	38/42 (90.5)	18/18 (100.0)	<0.001 (B1 vs. B2, B1 vs. B3)
Tumor thickness, μm, median (IQR)	305 (211.5–419.5)	1045 (446.3–1854.8)	4042.5 (2250–7243.8)	<0.001 (B1 vs. B2 vs. B3)
LNM, *n* (%)	1/63 (1.6)	2/42 (4.8)	10/18 (55.6)	<0.001 (B1 vs. B3, B2 vs. B3)

AVA	AVA‐S (*n* = 62)	AVA‐M (*n* = 40)	AVA‐L (*n* = 21)	*p*‐value
SEP, *n* (%)	26/62 (41.9)	35/40 (87.5)	21/21 (100.0)	<0.001 (AVA‐S vs. AVA‐M, AVA‐S vs. AVA‐L)
Tumor thickness, μm, median (IQR)	331 (210.3–537.5)	888.5 (378.5–1443.8)	3500 (2000–7342)	<0.001 (AVA‐S vs. AVA‐M vs. AVA‐L)
LNM, *n* (%)	1/62 (1.6)	6/40 (15.0)	6/21 (28.6)	<0.001 (AVA‐S vs. AVA‐M, AVA‐S vs. AVA‐L)

Abbreviations: AVA, avascular area; IQR, interquartile range; LNM, lymph node metastasis; NBI, narrowband imaging; SEP, subepithelial invasion.

## DISCUSSION

In this study, B2–B3 vessels and AVA‐M/L were identified as predictors of SEP in patients with pharyngeal cancer. Therefore, the use of ME‐NBI with the JES classification could predict the depth of superficial pharyngeal carcinoma. Moreover, there were significantly more B2–B3 vessels and findings of AVA‐M/L in the LNM‐positive group than in the LNM‐negative group, suggesting that the JES classification may be useful for predicting LNM.

It has also been reported that esophageal squamous cell carcinoma and pharyngeal squamous cell carcinoma are pathologically similar.^11^, ^12^ In terms of esophageal cancer, the JES classification shows that B1 vessels and an AVA‐S correspond to the invasion of the epithelium/lamina propria mucosa, B2 vessels, and AVA‐M to the invasion of the muscularis mucosae/upper third, and B3 vessels and AVA‐L to the invasion of the middle third.^8^, ^10^ In this study, the specificity and PPV of type B2–B3 vessels for SEP were 90.2% and 93.3%, respectively, and the specificity and PPV of AVA‐M/L for SEP were 87.8% and 91.8%. Therefore, there is a possibility of SEP in pharyngeal carcinoma with these findings. In particular, SEP was found in 18 of our cases with B3 vessels and 21 with AVA‐L, so these two features are important for a definitive diagnosis of SEP. Predicting the depth of laryngeal cancer prevents a positive vertical margin when resecting the tumor. Also, endoscopic submucosal dissection and endoscopic laryngopharyngeal surgery are basically not indicated for pharyngeal cancer that invades the deep muscle layer of bone. For these reasons, predicting the depth of laryngeal cancer has several benefits for minimally invasive treatments. The frequency of LNM based on the depth of invasion has been clarified in esophageal cancer. Cancers involving the epithelium/lamina propria mucosa have almost no risk of LNM, whereas the rate of LNM for cancers in the muscularis mucosae/upper third is reported to be about 10% and 20%–40% for cancers in the middle and lower thirds.^10^, ^13^, ^14^ Unlike esophageal cancer, the prevailing view is that the depth of pharyngeal cancer can be measured by substituting tumor thickness because the pharynx has no muscularis mucosae. The frequency of LNM of pharyngeal carcinoma reportedly increases when the tumor thickness is >1000 μm.^15^, ^16^, ^17^ In this study, 13 cases of pharyngeal carcinoma were found to have LNM, and all had a tumor thickness >1000 μm. However, tumor thickness and lymphovascular invasion related to LNM are only pathological findings, and resected specimens are necessary to obtain this information. Our LNM group had significantly more B2–B3 vessels and AVA‐M/L than our non‐LNM group, suggesting that in addition to tumor thickness, B vessels and AVA may be indicators of LNM. In particular, we found that the sensitivity and NPV of type B2 + B3 vessels for LNM were 93.2% and 95.2%, respectively. Therefore, the risk of LNM would be expected to be low if these findings were negative. There has been a report suggesting that submucosal invasion is more common when esophageal cancer has a protruding‐type morphology.^18^ In the present study, SEP was found in 25 (96.2%) of 26 pharyngeal carcinomas, which were significantly more likely to be the protruding type than the flat type. A combination of the JES classification and tumor morphology may improve the diagnostic accuracy of the depth of invasion in patients with pharyngeal carcinoma.

The findings of previous reports on ME‐NBI for pharyngeal cancer are summarized in Table 6.^17^, ^19^, ^20^, ^21^, ^22^, ^23^ Pharyngeal carcinoma with B2–B3 vessels has been reported to be significantly more likely to show SEP.^17^, ^21^ Furthermore, there are a few reports on the correlation between B vessels and tumor thickness.^22^, ^23^ In this study, the tumor thickness was significantly greater in the order of B1, B2, and B3 vessels, suggesting that the thickness of the tumor, which may be a risk factor for LNM, can be predicted by observing the microvascular pattern. In gastrointestinal cancers, if the depth of the tumor is shallower than the mucosal layer, there is almost no chance of LNM.^24^, ^25^, ^26^ Moreover, a previous study found that LNM is very rare in cases of pharyngeal cancer with a pathological diagnosis of CIS.^1^, ^17^ As in previous reports, we found almost no LNM in pharyngeal carcinoma diagnosed as CIS, which suggests a correlation between tumor depth and LNM. However, there are many cases of LNM in pharyngeal carcinoma that have invaded the subepithelial layer, so preoperative assessment of tumor depth is important when determining the treatment strategy. Katada et al. previously reported an association between JES classification and lymphatic invasion but did not separately analyze lymphatic invasion and LNM, treating them as the same factor.^22^ This study is one of the few to suggest a correlation between JES classification and LNM. Currently, fiberscopes for the head and neck are not yet equipped with a magnifying observation function and there are few reports of ME‐NBI of laryngeal cancer, so it is worth considering its usefulness.

**TABLE 6:** Summary of previous reports on magnifying endoscopy with narrowband imaging for pharyngeal cancer

First author	Year	Period	Study design	*n*	Tumor invasion	Type B vessels	Tumor thickness	Factors associated with SEP	Frequency of LNM/LVI
Fujii^19^	2010	2002–2006	Prospective	104	CIS: 75 (72.1%) SEP: 29 (27.9%)	N/A	CIS: 125–1000 μm SEP: 300–3500 μm (range)	Microvascular density, tumor thickness, tumor size	LNM(+): 1/104 (1.0%) ly(+): 1/104 (1.0%) v(+): 6/104 (5.8%)
Tateya^20^	2015	2007–2014	Retrospective	139	CIS: 101 (72.7%) SEP: 37 (26.6%) MP: 1 (0.7%)	N/A	N/A	Type 0‐I	ly(+): 3/139 (2.2%) v(+): 1/139 (0.7%)
Kikuchi^17^	2015	2008–2012	Retrospective	146	CIS: 41 (28.1%) SEP: 105 (71.9%)	B1: 128 (87.7%) B2: 14 (9.6%) B3: 4 (2.7%)	B1: 650.3 ± 577.4 μm B2: 720.9 ± 418.1 μm B3: 2256.5 ± 625.4 μm	Non B1 vessels	ly(+): 2/146 (1.4%) v(+): 3/146 (2.1%)
Eguchi^21^	2019	2016–2018	Retrospective	59	CIS: 21 (35.6%) CIS‐SEP: 13 (22.0%) SEP: 24 (40.7%) MP: 1 (1.7%)	B1: 26 (44.1%) B2: 28 (47.5%) B3: 4 (6.8%)	B1: 563 ± 614 μm B2: 1364 ± 1320 μm B3: 2825 ± 29,999 μm	Non B1 vessels	ly(+): 6/59 (10.2%) v(+): 7/59 (11.9%)
Katada^22^	2020	2006–2016	retrospective	92	CIS: 34 (37.0%) SEP: 58 (63.0%)	B1: 32 (47.8%) B2: 24 (35.8%) B3: 11 (16.4%)	B1: 580 ± 530 μm B2: 1264 ± 1238 μm B3: 4583 ± 3079 μm	White coat, main macroscopic type, macroscopic appearance, type B vessels	LNM(+): 7/92 (7.6%) ly(+): 12/92 (13.0%) v(+): 10/67 (14.9%)
Sunakawa, H^23^	2021	2011–2017	retrospective	219	CIS: 145 (66.2%) SEP: 74 (33.8%)	B1: 181 (82.6%) B2: 35 (16.0%) B3: 3 (1.4%)	B1: 275 μm B2/3:1325 μm (median)	B2/3 vessels	N/A

Abbreviations: CIS, carcinoma in situ; LNM, lymph node metastasis; LVI, lymphovascular invasion; ly, lymphatic invasion; N/A, not applicable; NBI, narrowband imaging; SEP, subepithelial invasion; v, venous invasion.

This study has several limitations. First, it had a retrospective design and was performed at a single institution. In this study, only 13 cases of pharyngeal carcinoma were found to have LNM, and this small sample size may have introduced a degree of bias. Only univariate analysis was performed to identify features that can predict LNM in pharyngeal cancer because of the small number of cases for comparison. Second, in this study, suspected LNM on preoperative imaging such as cervical ultrasonography, CT, and magnetic resonance imaging was confirmed by pathology, but pathology was not obtained for cases in which LNM was not suspected on these modalities. Preoperative imaging is not perfect for detecting LNM, and not pathologically confirming the status of LNM in all cases may have affected the results. Third, LNM was associated with histological findings including tumor thickness. Obtaining information about the risk of LNM by preoperative ME‐NBI may be useful for providing explanations to patients and deciding the treatment strategy before surgery, but the risk of LNM can be predicted based on histological findings, so ME‐NBI findings may not always be necessary. Fourth, the observation period was short, with LNM defined as occurring within 1 year of endoscopic diagnosis using a magnifying endoscope. The long‐term prognosis of LNM in pharyngeal carcinoma is important, but some patients in this study could not be followed for more than 1 year after treatment. Therefore, prospective multicenter studies are needed to investigate the long‐term course of metachronous LNM in the future.

## CONCLUSIONS

ME‐NBI using the JES classification could predict the depth of invasion in superficial pharyngeal carcinoma. The JES classification may contribute to the prediction of LNM, suggesting that it could serve as an alternative to tumor thickness.

## CONFLICT OF INTEREST

The authors declare no conflict of interest.

## FUNDING INFORMATION

None.
